# Cost-Effectiveness of Cardiac Biomarkers as Screening Test in Acute Chest Pain

**DOI:** 10.5681/jcvtr.2014.006

**Published:** 2014-03-21

**Authors:** Samad Shams-Vahdati, Zahra Vand-Rajavpour, Seyed-Pouya Paknezhad, Reza Piri, Elnaz Moghaddasi-Ghezeljeh, Saba Mirabolfathi, Mohammad Naghavi-Behzad

**Affiliations:** ^1^Department of Emergency Medicine, Imam Reza Hospital, Tabriz University of Medical Sciences, Tabriz, Iran; ^2^Students’ Research Committee, Tabriz University of Medical Sciences, Tabriz, Iran; ^3^Medical Philosophy and History Research Center, Tabriz University of Medical Sciences, Tabriz, Iran

**Keywords:** Cardiac Biomarkers, Creatine Kinase-MB, Myocardial Infarction, Acute Chest Pain

## Abstract

***Introduction:*** Acute chest pain is an important and frequently occurring symptom in patients. Chest pain is often a sign of ischemic heart disease. Associated findings of electrocardiograph (ECG) are rather heterogeneous, and traditional cardiac biomarkers such as Creatine Kinase-MB (CK-MB) suffer from low cardiac specificity and sensitivity. In this study cost effectiveness of cardiac biomarkers single quantitative measurement was examined.

***Methods:*** The present descriptive-analytic study conducted on patients who were asked for troponin I and CK-MB. All patients who referred to Emergency unit of Tabriz Imam Reza educational-medical center during January 2012 to July the 2013 were included in study. All patients included in the study were documented in terms of age, sex, working shift of referring, main complaint of patient, symptoms in referring, ECG findings, and results of troponin I and CK-MB tests.

***Results:*** In this study, 2900 patients were studied including 1440 (49.7%) males and 1460 (50.3%) females. Mean age of patients was 62.91 (SD=14.36). Of all patients 1880 (64.8%) of patients referred during 8 a.m. to 8 p.m. and 1020 (35.2%) patients were referred during 8 p.m. to 8 a.m. The sensitivity of cardiac biomarkers’ test in diagnosing Acute Coronary Syndrome (ACS) disease was calculated as 44.8% and its specificity was 86.6%. For diagnosing Acute Myocardial Infarction (AMI), sensitivity of cardiac biomarkers’ test was 72.2% and its specificity was 86%. None of patients who were finally underwent unstable angina diagnosis showed increase in cardiac enzymes.

***Conclusion:*** In conclusion, cardiac biomarkers can be used for screening acute chest pains, also cost effectiveness of cardiac biomarkers, appropriate specificity and sensitivity can guarantee their usefulness in emergency room.

## Introduction

Every year, over 1.5 million patients are admitted to the hospitals after presenting to the emergency department with acute chest pain.^1,2^ Only a small percentage of these admitted persons have acute coronary syndromes and the vast majority of patients are discharged with non-cardiac diagnoses.^3^ Acute chest pain is an important and frequently occurring symptom in patients.^4,5^ Chest pain is often a sign of ischemic heart disease, although gender, age and comorbidity may modify how acute Coronary Heart Disease (CHD) presents itself within the individual patient. Acute chest pain may indicate a potentially life threatening situation, but it is also commonly acknowledged that a wide variety of differential diagnosis exists, many with lower health impact and less serious potential.^6,7^ The acute coronary syndrome is a high-risk phase for patients with CHD mainly defined by clinical symptoms.^8^ Associated findings of electrocardiograph (ECG) are rather heterogeneous, and traditional cardiac biomarkers such as Creatine Kinase-MB (CK-MB) suffer from low cardiac specificity and sensitivity.^9^ CK-MB levels, along with total CK, are tested in persons who have chest pain to diagnose whether they have had a heart attack. Since a high total CK could indicate damage to either the heart or other muscles, CK-MB helps to distinguish between these two sources.^10^

Cardiac biomarkers were first developed for assisting the diagnosis of cardiac events, especially Acute Myocardial Infarction (AMI). The discoveries of other cardiac biomarkers, the better understanding of cardiac disease process and the advancement in detection technology has pushed the applications of cardiac biomarkers beyond the ‘diagnosis’ boundary. Not only the measurements of cardiac biomarkers are more sensitive, but also the applications have now covered staging of cardiac disease, timing of cardiac events and prognostication.^11-14^ Troponin, a complex of three contractile regulatory proteins, troponin C, T and I, controls the calcium-mediated interactions between actin and myosin in cardiac and skeletal muscles. Troponin-I and T are specific to cardiac muscles, unlike troponin-C which is associated with both cardiac and skeletal muscles. Hence, troponin-C is not used in the diagnosis of myocardial damage.^15^ Test systems for cardiac troponin T and troponin I (CTnI) provide the highest cardiac specificity and analytical sensitivity for the detection of myocardial injury.^16-18^ These markers allow early identification of patients with AMI and in one-third of patients with unstable angina (in spite of normal CK enzyme activity), a minor increase in troponin related to myocardial injury is detectable. Several studies have documented the superior prognostic value of the troponins for early and safe risk stratification of patients with acute chest pain.^19,20^ Depending on the cardiac biomarker, it takes between 2 to 24 hours for the level to increase in the blood. Additionally, determining the levels of cardiac markers in the laboratory - like many other lab measurements takes substantial time. Cardiac markers are therefore not useful in diagnosing a Myocardial Infarction (MI) in the acute phase. The clinical presentation and results from an ECG are more appropriate in the acute situation.^21-23^

It is recommended that a whole blood point-of-care analysis of multiple cardiac markers that provides excellent clinical sensitivity and specificity for the detection of AMI^24^, but it is mentioned that serial enzyme measurements as one of the three criteria for diagnosis, the other two being ECG changes and clinical features such as chest pain.^25^

Also it is concluded in many studies that qualitative (positive/negative) and quantitative point-of-care test devices for troponin I as well troponin T have been shown to deliver reliable results.^26,27^ While on the other hand other studies quantitative measurement is recommended.^28^

In a study by Hoekstra et al. about efficacy of CK-MB in predicting an risk for complications of myocardial ischemia in patients, it was concluded that CK-MB measurements can help risk-stratify Emergency Department (ED) chest pain patients whose initial ECGs are without diagnostic ST-segment elevation.^29^

According to mentioned studies, cardiac biomarkers can be used for screening acute chest pains. In this study cost effectiveness of cardiac biomarkers single quantitative measurement was examined.

## Materials and methods

The present descriptive-analytic study was conducted on patients who were checked for CTnI and CK-MB. All patients who referred to the emergency department of Tabriz Imam Reza Educational-Medical center during January 2012 to July 2013 and those for CTnI and CK-MB levels by professors in medicine of emergency department or by other consulting services were included to the study. Patients who did not permit for blood sampling and patients under 13 years of age (since the hospital does not accept under 13-year-old patients) were excluded.

All patients included in the study were documented in terms of age, sex, working shift of referring, chief complaint of patient, symptoms in referring, ECG findings, and results of CTnI and CK-MB tests. Data obtained from the study were statistically analyzed using statistical software of SPSS.15 and by descriptive statistical methods (frequency, percentage, and mean±standard deviation) and mean difference tests for independent groups including Independent Samples T-Test, one way ANOVA, Repented Measurement of ANOVA and Chi-square with Fisher’s Exact Test. In this study, P value less than 0.05 was statistically considered significant. Normality of data distribution was evaluated by Kolmogorov-Smirnov test. All qualitative and quantitative data of analysis were described and then the relation between patient’s complaint and enzymes, patient’s complaint and final diagnosis, symptoms identified during physical examination, enzymes, symptoms, and diagnosis were determined.

## Results

In this study, 2900 patients were studied including 1440 (49.7%) males and 1460 (50.3%) females. Mean age of patients was 62.91 years old (SD=14.36) which was 59.9 in men and 65.8 in women. Of all patients, 1880 (64.8%) of patients referred during 8 a.m. to 8 p.m. and 1020 (35.2%) patients were referred during 8 p.m. to 8 a.m.

Chest pain variants including simultaneous occurrence of atypical chest pain, dyspnea and dizziness were the most prevalent complaints with 18.6% and dyspnea with 14.5% was in the second rank. Epigastric pain had the least prevalence of 3.45%. About patients who were asked for cardiac biomarkers, 1140 (39.3%) patients had no findings in clinical examinations. In clinical examinations of referred patients, abnormal findings of lung auscultation were the most prevalent finding (21.4%). ECG was normal in 40.7% of patients and invert T was the most prevalent abnormal finding in ECG (14.5%). Serial ECG was taken for 29% of patients. Cardiac enzymes were positive for 10.3% of patients.

Of patients who referred with loss of consciousness 31.6%, and 19% of those who refereed with dyspnea, showed increase in cardiac enzymes. None of patients referred with complaint of just chest pain showed increase in enzymes. There was increase in cardiac enzymes in 28.6% of patients with abnormal findings of heart auscultation and in 22.6% of patients with abnormal findings of lung auscultation. The most frequent reason for asking cardiac biomarker tests was non-cardiac complaints.

Of all patients, 757 (26.1%) patients were hospitalized due to cardiac problems and 43.45% of them were hospitalized with diagnosis of problems irrelevant to cardiac diseases and others were discharged. In this study, 86.7% of patients had increase in AMI cardiac biomarkers and the others had lung diseases like pulmonary thromboemboli. Cardiac issues were diagnosed in 12.3% of patients with normal cardiac enzymes.

The sensitivity of cardiac biomarkers’ test in diagnosing Acute Coronary Syndrome (ACS) disease was calculated as 44.8% and its specificity was 86.6%. For diagnosing AMI, sensitivity of cardiac biomarkers’ test was 72.2% and its specificity was 86%. None of patients who were finally given unstable angina diagnosis showed increase in cardiac enzymes.

## Discussion

In our study, serial testing in 2900 consecutive patients with chest pain established that cardiac biomarkers at the time of admission, as compared with other clinical and para-clinical findings provided substantially improved levels of diagnostic accuracy and discrimination for diagnosis and ruling out MI and ACS.

In a prospective study of diagnostic and prognostic value of rapid bedside troponin T and troponin I testing for early triage in the emergency room, it was concluded that less than half of patients (16%) had at least one positive bedside-test result for cardiac biomarkers, or (22%) had at least one positive test for cardiac biomarkers^26^, as results of present study declares the same findings. Also in this study, ECG ST-T alterations other than ST-segment elevations were found in 355 patients (46%); 158 patients had ST-segment depressions, and 197 patients had T-wave inversions. In 87 patients (11%), the electrocardiogram was non-diagnostic (paced rhythm, bundle-branch block); 23 of these patients had MI.^26^

In another study about incidence of prognostically important myocardial damage in patients discharged from ED it was concluded that 30% of patients had non cardiac chest pain^30^, which is similar to what was concluded in present study (26.1%).

In a study about diagnoses of patients admitted with acute chest pain but without MI, it was concluded that pulmonary embolism, gastro-esophageal diseases and chest-wall syndromes should be paid special attention because of their prominent sign and symptoms. A careful physical examination of the chest wall and an upper endoscopy seems to be the most cost-beneficial examination to employ in this subset.^31^

In the present study, the sensitivity of cardiac biomarkers’ test in diagnosing ACS disease was calculated as 44.8% and its specificity was 86.6%. For diagnosing AMI, sensitivity of cardiac biomarkers’ test was 72.2% and its specificity was 86%. However in another study, examining myoglobin, CK-MB, and cardiac troponin I for diagnosis of acute MI, CTnI had a clinical sensitivity of 70% as compared to 21% and 18% for myoglobin and CK-MB, respectively. The clinical specificity of CTnI for non-AMI patients was equivalent to CK-MB and significantly higher than for myoglobin. The clinical efficiency of CTnI for all samples was better than either CK-MB or myoglobin.^32^ In another study about comparing cardiac biomarkers, it was shown that criteria based on CTnI should improve the accuracy of retrospective diagnoses of AMI because CTnI has been shown to be highly specific for myocardial damage and to have sensitivity comparable with that of CK-MB isoenzyme for detecting cardiac injury.^33^

In another study of cardiac biomarkers as predictor of major cardiac events in ED patients with acute chest pain, it was concluded that the sensitivity and specificity of CK-MB for diagnosing AMI was respectively 96% and 97% but this values for CTnI were 75% and 97% .^34^ Also a study about efficacy of serial cardiac bio marker sampling it was concluded that rule-out of AMI requires serial collection and testing of blood for cardiac markers. When an early marker such as myoglobin is used, acute myocardial necrosis can be effectively ruled out within 6–9 h after ED presentation.^35^ This study is similar to present study’s findings because single measurement of cardiac markers lacked enough specificity and sensitivity.

In another study about comparing directly serial versus single time-point measurements of CTnI, it was shown that single measurement of CTnl on any of the first 4 days, particularly on day 4 after onset of AMI, gives a good estimation of infarct size. Single-point measurements are convenient, easy and inexpensive and may gain clinical acceptance because they are as effective as serial measurements^36^; while our study did not prove that.

A study rapid measurement of whole blood myoglobin, CK-MB, and CTnT by the triage cardiac panel for detection of MI has shown that that neither serial nor parallel analysis of the multiple markers provided increased sensitivity or specificity for detection or ruling out of MI^24^; this is in contrast with present study’s findings.

The triage cardiac panel and device (both Food and Drug Administration approved) offer clinicians quantitative whole blood analysis of multiple cardiac markers and can serve as a point-of-care testing device in the emergency room, the coronary care unit, or at the bedside^24^, which is similar to findings of present study.

Despite findings of the present study, in a study of emergency room triage of patients with acute chest pain by means of rapid testing for CTnI, it was concluded that qualitative positive/negative bedside tests for CTnI and cTnT that have demonstrated high sensitivity for the detection of acute MI after 6 hours, with negative results was associated with low risk and safe discharge of patients with an episode of acute chest pain.^26^

In conclusion, cardiac biomarkers can be used for screening acute chest pains; also cost effectiveness of cardiac biomarkers, appropriate specificity and sensitivity can guarantee their usefulness in emergency room. Further investigation on more patients and also with examining other biomarkers (such as inflammatory) biomarkers are suggested.

## Ethical issues

Study protocol was approved by the Ethics Committee of TUMS, which was in compliance with Helsinki Declaration.

## Competing interests

The authors declare that there is no conflict of interests.
